# Diretrizes, Posicionamentos e Normatizações: Documentos de Auxílio à Prática Médica

**DOI:** 10.36660/abc.20220001

**Published:** 2022-08-25

**Authors:** Antônio Carlos Sobral Sousa, Harry Corrêa, Bruno Nascimento, Aurora Castro Issa, Marcelo Luiz Campos Vieira, Brivaldo Markman

**Affiliations:** 1 Universidade Federal de Sergipe Aracaju SE Brasil Universidade Federal de Sergipe – UFS, Aracaju, SE – Brasil; 2 Instituto de Cardiologia de Santa Catarina São José SC Brasil Instituto de Cardiologia de Santa Catarina, São José, SC – Brasil; 3 Universidade do Sul de Santa Catarina Palhoça SC Brasil Universidade do Sul de Santa Catarina – UNISUL, Palhoça, SC – Brasil; 4 Hospital das Clínicas Universidade Federal de Minas Gerais Belo Horizonte MG Brasil Hospital das Clínicas da Universidade Federal de Minas Gerais, Belo Horizonte, MG – Brasil; 5 Instituto Nacional de Cardiologia Rio de Janeiro RJ Brasil Instituto Nacional de Cardiologia, Rio de Janeiro, RJ – Brasil; 6 Universidade de São Paulo Instituto do Coração São Paulo SP Brasil Universidade de São Paulo Instituto do Coração, São Paulo, SP – Brasil; 7 Hospital Israelita Albert Einstein São Paulo SP Brasil Hospital Israelita Albert Einstein, São Paulo, SP – Brasil; 8 Universidade Federal de Pernambuco Recife PE Brasil Universidade Federal de Pernambuco, Recife, PE – Brasil

**Keywords:** Guia de Prática Clínica, Guia, Doenças Cardiovasculares

O ato médico, que deve ser compartilhado, é fundamentado em dois pilares, o intelectivo, incapaz de ser padronizado, porque depende da capacidade cognitiva do profissional na tomada da decisão e o técnico que depende da formação, aperfeiçoamento e atualizações, podendo, portanto, ser regulado por diretrizes de prática clínica (DPC). Estas constituem ferramentas importantes, especialmente em uma área tão complexa e em rápida mudança como a cardiologia, objetivando: melhorar a qualidade do atendimento, baseado na melhor evidência disponível e reduzir a disparidade de condutas para o mesmo tipo de situação clínica.^1 , 2^

Ressaltando-se que a sua aderência varia muito, e que alguns médicos têm preocupações de que estes instrumentos caracterizem uma rígida ou simplificada prática da medicina.^3^ Portanto, a implementação apropriada de DPC constitui grande interesse para organizações nacionais, sociedades profissionais, prestadores de cuidados à saúde, responsáveis políticos, judicialização da Medicina, pacientes e o público em geral. Dada a importância do tema, várias ferramentas têm sido desenvolvidas para avaliar a credibilidade das diretrizes existentes,^4^ bem como orientações passo a passo para a concretização de um documento prático e confiável.^5^

A Sociedade Brasileira de Cardiologia (SBC) tem publicado, sistematicamente, desde 1992, diretrizes sobre os temas mais relevantes da especialidade.^6^ Todavia, tem sido registrado falta de discernimento de três conceitos importantes,^7^ nesta pretensão, por parte dos departamentos que compõem a SBC: a) diretriz é o termo que deve ser reservado para o documento que sumariza, formalmente, evidências nas áreas de diagnóstico e terapêutica de patologias; b) posicionamento (ou orientação clínica), que deve ser utilizado para impressos oficiais que fornecem aconselhamento especializado sobre desafios na condução de pacientes e, c) normatização (ou comunicação), que, por sua vez, deve ser empregado para os manuscritos que informam a metodologia laboratorial e definições de desfecho clínico.

Torna-se imperativo que os documentos emitidos pela SBC se apresentem com titulação e fundamentação adequadas para que seja evitada, por parte do leitor, confusão na diferenciação dos termos e, consequentemente, desinteresse na leitura dos mesmos.

Portanto, o objetivo principal desta publicação é o de estabelecer de forma simplificada e objetiva o significado destas terminologias, visando padronizar a emissão de diretrizes, comunicações e orientações por parte da SBC.

## Diretrizes de prática clínica

As DPC são constituídas de afirmações sistematicamente desenvolvidas e são projetadas para apoiar os processos de tomada de decisão na assistência ao paciente, em condições específicas.^8^ Ao contrário de um documento de orientação, uma diretriz aborda um tópico em que há evidências de moderada a alta qualidade, geralmente provenientes de ensaios randomizados com número satisfatório de integrantes, para possibilitar as práticas clínicas mais adequadas.

Na sua elaboração é utilizado um processo para resumir as evidências e fornecer um método padronizado para expressar os graus de recomendações com os seus respectivos níveis de evidências. Para que a diretriz seja confiável, é prudente que sejam observados os seguintes critérios: a) ser baseada em revisões sistemáticas da literatura; b) ser desenvolvida por um painel multidisciplinar e experiente de especialistas; c) considerar os valores e preferências dos pacientes, assim como os seus subgrupos; d) ser baseada em um processo explícito e transparente que minimiza distorções, vieses e conflitos de interesse; e) fornecer uma explicação clara das relações entre as opções de cuidados alternativos e desfechos clínicos e, f) ser atualizada quando novas importantes evidências justificarem modificações de recomendações.^9^

As diretrizes podem melhorar desfechos clínicos, todavia, elas apresentam adesões variáveis.^10^ Elas raramente abordam prática médica onde as evidências são escassas. Portanto, torna-se necessária a utilização de estratégias inovadoras para facilitar a disseminação desses documentos. Vale ressaltar que as DPC não são livros de receitas, já que a maioria destes documentos apresenta limitações em sua disponibilidade e aplicabilidade no contexto do nível de evidência das recomendações, já que apenas uma pequena percentagem está embasada em estudos clínicos randomizados.^11^ Consequentemente, se faz necessária a atualização frequente destas diretrizes para a incorporação de evidências mais robustas que eventualmente surgirem.

### Documento de posicionamento

Estes documentos visam abordar um determinado tópico (diagnóstico, terapêutico ou laboratorial) de reconhecido interesse clínico, para o qual não existem (ou é improvável que venha a acontecer) evidências de qualidade substancial, notadamente aquelas oriundas de ensaios clínicos randomizados. Tais documentos são complementares às diretrizes e são elaborados por uma equipe de profissionais com experiência estabelecida no tema.

Como exemplo, poderíamos citar o uso dos anticoagulantes diretos em pacientes gestantes.^11^ Em geral, as orientações contidas nestes documentos permanecem ancoradas nas melhores evidências disponíveis; todavia, incorporam, frequentemente, a opinião pessoal dos especialistas.

### Documento de normatização

Estes dispositivos diferem dos acima relacionados uma vez que abordam tópicos primariamente direcionados para a padronização de práticas clínicas, laboratoriais e de metodologias de pesquisa. Como exemplo, poderíamos citar a comunicação do Subcomitê de Controle de Anticoagulação da Sociedade Internacional de Trombose e Hemostasia para medir a atividade anticoagulante dos inibidores do fator Xa.^12^ Portanto, trata-se de ferramenta útil à disposição dos departamentos da SBC.

Concluindo, o movimento em direção aos cuidados de saúde baseados em evidências vem ganhando terreno rapidamente nos últimos anos, motivado por clínicos, políticos e gestores, preocupados com a qualidade, consistência e custos da assistência médica.

Assim, os documentos acima mencionados, baseados nas melhores práticas padronizadas, desde que redigidos de forma prática e objetiva, podem ser capazes de promover melhorias na qualidade e consistência dos cuidados com a saúde. Garantir a aplicabilidade e a implementação dessas recomendações dependerá da aceitabilidade do paciente, da disponibilidade do procedimento, da experiência necessária no contexto específico, além do seu impacto quando colocadas em prática.^13^
