# Supplementary material for: Diretrizes, Posicionamentos e Normatizações: Documentos de Auxílio à Prática Médica
Source: Arq Bras Cardiol. 2022 Aug 25;119(3):496–8. [Article in Portuguese] doi: 10.36660/abc.20220001 (PMC9438544; doi:10.36660/abc.20220001)
Supplement: Translation

# Translation: Guidelines, Position Statements, and Standardizations: Documents to Assist Medical Practice

**Authors:** Antônio Carlos Sobral Sousa, Harry Corrêa, Bruno Nascimento, Aurora Castro Issa, Marcelo Luiz Campos Vieira, Brivaldo Markman

**Affiliations:** 1 Brazil Universidade Federal de Sergipe – UFS, Aracaju, SE – Brazil; 2 Brazil Instituto de Cardiologia de Santa Catarina, São José, SC – Brazil; 3 Brazil Universidade do Sul de Santa Catarina – UNISUL, Palhoça, SC – Brazil; 4 Brazil Hospital das Clínicas da Universidade Federal de Minas Gerais, Belo Horizonte, MG – Brazil; 5 Brazil Instituto Nacional de Cardiologia, Rio de Janeiro, RJ – Brazil; 6 Brazil Universidade de São Paulo Instituto do Coração, São Paulo, SP – Brazil; 7 Brazil Hospital Israelita Albert Einstein, São Paulo, SP – Brazil; 8 Brazil Universidade Federal de Pernambuco, Recife, PE – Brazil

**Keywords:** Practice Guideline, Guideline, Cardiovascular Diseases

Medical practice, which must be shared, is based on two pillars, the intellectual one, which cannot being standardized because it depends on the cognitive capacity of the professional when making decisions, and the technical one, which depends on training, improvement, and updates and which can, therefore, be regulated by clinical practice guidelines (CPG). CPG are important tools, especially in an area as complex and rapidly changing as cardiology, and they have the following objectives: to improve the quality of care based on the best available evidence and to reduce the disparity of medical conduct for the same type of clinical situation.^1 , 2^

It is worth underscoring that adherence to CPG varies greatly and that some physicians are concerned that these instruments represent rigid or simplified practice of medicine.^3^ Therefore, the proper implementation of CPG is of great interest to national organizations, professional societies, healthcare providers, policymakers, the judicialization of Medicine, patients, and the general public. Given the importance of the topic, several tools have been developed to evaluate the credibility of existing guidelines,^4^ as well as step-by-step guidance on how to produce practical and reliable documents.^5^

Since 1992, the Brazilian Society of Cardiology (SBC) has systematically published guidelines on the most relevant topics in the specialty.^6^ Nonetheless, a lack of discernment has been registered in relation to three important concepts,^7^ in this effort, on the part of the departments that compose the SBC: a) guideline is the term that should be reserved for documents that formally summarize evidence in the areas of disease diagnosis and therapy; b) position statement (or clinical guidance) should be used for official publications that provide expert advice on challenges in patient management; and c) standardization (or communication), in turn, should be used for manuscripts that inform laboratory methodology and definitions of clinical outcomes.

It is imperative for the documents published by the SBC to be presented with appropriate titles and foundations in order to avoid confusion on the part of the reader in differentiating these terms and consequent lack of interest in reading them.

Therefore, the main objective of this publication is to establish, in a simplified and objective manner, the meaning of these terms, aiming to standardize the publication of guidelines, communications, and position statements by the SBC.

## Clinical practice guidelines

CPG are made up of systematically developed statements, and they are designed to support decision-making processes in patient care, under specific conditions.^8^ Unlike documents that provides guidance, guidelines address a topic where there is moderate to high quality evidence, generally from randomized trials with a satisfactory number of participants, to make the most appropriate clinical practice possible.

In drafting them, a process is used to summarize the evidence and provide a standardized method to express the classes of recommendations with their respective levels of evidence. For a guideline to be reliable, it is advisable to observe the following criteria: a) be based on systematic reviews of the literature; b) be developed by a multidisciplinary and experienced panel of experts; c) consider the values and preferences of patients, as well as their subgroups; d) be based on an explicit and transparent process that minimizes distortions, biases, and conflicts of interest; e) provide a clear explanation of the relationships between alternative care options and clinical outcomes, and f) be updated when important new evidence warrants changes to recommendations.^9^

Guidelines can improve clinical outcomes; nevertheless, adherence to them varies.^10^ They rarely address medical practice where evidence is scarce. Therefore, it is necessary to use innovative strategies to facilitate the dissemination of these documents. It is worth underscoring that CPG are not recipe books, given that most of them have limitations in their availability and applicability in the context of the level of evidence of the recommendations, as only a small percentage is based on randomized clinical trials.^11^ Consequently, it is necessary to frequently update these guidelines in order to incorporate more robust evidence that eventually emerges.

### Positioning documents

These documents aim to address a determined diagnostic, therapeutic, or laboratory topic of recognized clinical interest, for which evidence of substantial quality, notably evidence from randomized clinical trials either does not exist or is unlikely to be produced. These documents are complementary to the guidelines, and they are prepared by a team of professionals with established experience on the topic.

As an example, we may cite the use of direct anticoagulants in pregnant patients.^11^ In general, the guidance contained in these documents continues to be anchored in the best available evidence; nonetheless, they frequently incorporate the personal opinion of experts.

### Standardization documents

These documents differ from those listed above, insofar as they address topics primarily aimed at standardizing clinical and laboratory practices and research methodologies. We may cite, as an example, the report of the Subcommittee on Control of Anticoagulation of the International Society of Thrombosis and Haemostasis to measure the anticoagulant activity of factor Xa inhibitors.^12^ It is, therefore, a useful tool that is available to departments of the SBC.

In conclusion, the movement toward evidence-based healthcare has been rapidly gaining ground in recent years, driven by clinicians, policymakers, and managers who are concerned about the quality, consistency, and costs of healthcare.

Accordingly, these documents, based on standardized best practices, provided that they are written in a practical and objective manner, are able to promote improvements in the quality and consistency of healthcare. Guaranteeing the applicability and implementation of these recommendations will depend on the extent to which patients accept them, the availability of procedures, the experience required in the specific context, and their impact when put into practice.^13^
